# UBE2T Contributes to the Prognosis of Esophageal Squamous Cell Carcinoma

**DOI:** 10.3389/pore.2021.632531

**Published:** 2021-04-09

**Authors:** Xiaoyuan Wang, Yang Liu, Xue Leng, Kui Cao, Wentao Sun, Jinhong Zhu, Jianqun Ma

**Affiliations:** ^1^ Department of Thoracic Surgery, Harbin Medical University Cancer Hospital, Harbin, China; ^2^ Department of Oncology, Harbin Medical University Cancer Hospital, Harbin, China; ^3^ Department of Radiology, Harbin Medical University Cancer Hospital, Harbin, China; ^4^ Department of Clinical Laboratory, Biobank, Harbin Medical University Cancer Hospital, Harbin, China

**Keywords:** UBE2T, ESCC, prognosis, TCGA, immunohistochemistry

## Abstract

**Background:** The ubiquitin-conjugating enzyme E2 T (UBE2T) has been shown to contribute to several types of cancer. However, no publication has reported its implication in esophageal squamous cell cancer (ESCC).

**Methods:** We explored several public databases, including The Cancer Genome Atlas (TCGA), Oncomine, and gene expression Omnibus (GEO). Gene Ontology (GO), Kyoto Encyclopedia of Genes and Genomes (KEGG) pathway enrichment analysis, and gene set enrichment analysis (GSEA) were adopted to explore involved signaling pathways. We used R software to develop prognostic gene signatures with the LASSO and stepwise Cox regression analysis, separately. Immunohistochemistry staining was performed to detect UBE2T in 90 ESCC patients, followed by survival analysis. We also used an R package pRRophetic to evaluate chemotherapy sensitivity for the TCGA–ESCC cohort.

**Results:** We found significantly increased *UBE2T* transcript levels and DNA copy numbers in ESCC tissues. UBE2T was associated with the p53 signaling pathway, cell cycle, Fanconi anemia pathway, and DNA replication, as indicated by Go, KEGG pathway enrichment analysis. These pathways were also upregulated in ESCC. The prognostic signatures with UBE2T-associated genes could stratify ESCC patients into low- and high-risk groups with significantly different overall survival in the TCGA–ESCC cohort. We also validated the association of UBE2T with unfavorable survival in 90 ESCC patients recruited for this study. Moreover, we found that the low-risk group was significantly more sensitive to chemotherapy than the high-risk group.

**Conclusions:** UBE2T is involved in the development of ESCC, and gene signatures derived from UBE2T-associated genes are predictive of prognosis in ESCC.

## Introduction

Esophageal cancer (EC) is one of the major health problems, which ranks 7th for incidence (572,000 new cases), but 6th for mortality (509,000 deaths) worldwide [1]. EC is mainly composed of esophageal squamous cell cancer (ESCC) and adenocarcinoma. China is located in the so-called esophageal cancer Belt, the highest-risk region. ESCC is the predominant histology type in China, accounting for over 90% of all EC cases [1, 2]. It was estimated that for every 20 patients who die from cancer, one dies from EC [1]. The disproportionally high mortality rate of EC is partially due to late diagnosis. Nearly half of the EC patients present with unresectable or metastatic lesions at the time of diagnosis. Patients with early diseases screened by endoscopy may greatly benefit from surgery. However, patients with locally advanced diseases usually rely on chemotherapy, radiotherapy, or adjuvant chemotherapy following the operation. Concurrent radiation and chemotherapy are often essential to prolong the lifespan of patients with advanced or metastatic EC [3]. The overall 5-year survival of EC has been modestly improved from less than 5% in the 1960s to about 20% in the past decade [4], but remains far from satisfying. Comprehensively understanding the molecular mechanisms of ESCC may accelerate novel therapy development and the discovery of biomarkers for early diagnosis and prognosis.

The ubiquitin-conjugating enzyme E2 T (UBE2T) is a member of the UBE2 family. This enzyme catalyzes the ubiquitination of proteins, a posttranslational modification. Ubiquitination modification takes place sequentially through the following steps: the activation of the ubiquitin-activating enzymes (E1s), the binding of E2s, and the binding of ubiquitin-ligating enzymes (E3s) [5, 6]. Ubiquitination plays an indispensable role in proteasome-mediated protein degradation. Alternatively, ubiquitination may alter the location, function, and activity of proteins, thereby affecting the cell cycle and regulating cancer-related processes such as DNA repair and inflammation [7–9]. UBE2T was first identified in Fanconi Anemia (FA), which is responsible for DNA damage repair as a critical member of the FA pathway. UBE2T also participates in the ubiquitination of target proteins by coupling with specific E3, leading to the breakdown of substrate molecules through the proteasome-mediated protein degradation pathway [10]. Moreover, increasing evidence has shown that UBE2T is involved in the carcinogenesis of different types of tumors, including lung cancer [11], gastric cancer [12], hepatocellular carcinoma [13], nasopharyngeal [14], osteosarcoma [15], and prostate cancer [16]. Elevated expression levels of UBE2T were observed in various malignant tumor tissues, which seem to relate to tumor size, the degree of malignancy, metastasis, and poor prognosis of tumor patients [11, 12, 14, 15]. Collectively, these results suggest that UBE2T may be a therapeutic target for cancer. However, the implications of UBE2T in ESCC have not been reported to date. Due to the general significance of UBE2T in tumors, we investigated the contributing role of UBE2T in ESCC by performing bioinformatics analysis, and UBE2T immunohistochemistry (IHC) staining on ESCC samples was also conducted. We observed significantly upregulated UBE2T in ESCC in comparison to adjacent non-cancerous tissues. UBE2T was associated with clinical outcomes in ESCC. Moreover, we explored the underlying mechanisms by which UBE2T might contribute to the development of ESCC. Finally, we used the LASSO and stepwise Cox regression algorithm to construct multi-gene prognostic signatures in ESCC, based on *UBE2T* and associated genes.

## Materials and Methods

### Data Collection

We first compared *UBE2T* expression between ESCC and normal tissues. *UBE2T* mRNA expression data in cancerous and adjacent non-cancer/normal esophageal tissues/blood were retrieved from the Oncomine (www.oncomine.org), TCGA (https://cancergenome.nih.gov/), and gene expression omnibus (GEO) databases (http://www.ncbi.nlm.nih.gov/geo/). Because the TCGA-ESCA project involved tumor samples of different histology types, data on 10 normal tissues and 80 squamous cell neoplasms were extracted for further analysis. Three GEO data series with GPL570 (HG-133_Plus_2) Affymetrix Human Genome U133 Plus 2.0 Array were collected, including GSE100942 (5 pairs of ESCC tumor and adjacent non-tumor tissues), GSE17351 (5 pairs of ESCC tumor and normal tissues), and GSE45670 (38 ESCC tumor tissues and normal esophageal epithelia).

### Patients and Samples

In this study, we retrospectively obtained formalin-fixed paraffin-embedded tissue specimens from 90 patients with ESCC, who underwent surgery in the Harbin medical university cancer hospital from January 2012 to December 2012. Characteristics of patients were listed in the Supplementary Table. The clinical data were retrieved from electronic medical records, and the patients did not receive any anticancer treatments before surgery. All patients were followed up for more than five years from the day of the surgical operation. This study was approved by the Ethics Committee of Harbin Medical University Cancer Hospital, and written consent was provided by patients or relatives before participating in this study. Clinical staging of patients with ESCC was determined based on the 2017 NCCN guidelines for esophageal cancer staging criteria.

### Immunohistochemistry Staining

We used a paraffin slicing machine (Leica, Germany) to cut paraffin slices at about 4 microns. The slices were baked in a 67°C oven for 2 h and deparaffinized in xylene and rehydrated in graded ethanol, then boiled in citrate buffer (pH 6.0) for 3 min at 100°C and cooled naturally to room temperature. We immersed the sections in 3% H_2_O_2_ for 10 min to block the endogenous peroxidase and washed with PBS. Sections were further incubated with anti-UBE2T antibody (cat#: ab154022, Abcam, Cambridge, MA, United States) overnight at 4°C in a humidified container. The next day, the sections were washed with PBS three times and then incubated with a horseradish peroxidase-labeled secondary antibody (cat#: ab205718, Abcam, Cambridge, MA, United States) for 1 h at room temperature. Sections were washed with PBS three times again. A drop of DAB (50:1, Novus Biologicals, Centennial, CO, United States) was added to every section, which was observed under microscopy for timely termination. Hematoxylin was used to counterstain sections briefly, which were observed under a microscope. Finally, sections were dehydrated in ethanol and sealed with neutral resin. In the case of negative control, the primary antibody was omitted. The score of relative staining intensity was 0, 1, 2, 3, and 4. Tissue with score ≤1 or ≥2 was defined as low and high expression, respectively.

### Acquirement and Analysis of Coexpression Genes of *UBE2T* in ESCC

We obtained genes highly positively correlated with *UBE2T* from a study by Su and colleagues [17] from the Oncomine database. Gene Ontology (GO) and Kyoto Encyclopedia of Genes and Genomes (KEGG) pathway analysis were performed to help understand the potential functions of these genes. The functional annotation of GO, based on biological process (BP), cellular component (CC), and molecular function (MF), was conducted using the open-access WebGestalt tool (http://www.webgestalt.org). The same tool was also adopted to perform KEGG pathway analysis to found biological pathways in which these genes were enriched.

We generated a protein-protein interaction (PPI) network using the Search Tool for the Retrieval of Interacting Genes (STRING; version 9.0; http://string-db.org), with a combined score >0.4. The interaction data was imported into and analyzed in Cytoscape (version 3.4.0), an open-source bioinformatics software platform commonly used to visualize molecular interaction networks. We also took advantage of a plugin Molecular Complex Detection (MCODE) (version 1.4.2) of Cytoscape to dissect the most significant modules in the PPI networks. Parameters were preset at MCODE scores >5, degree cut-off = 2, node score cut-off = 0.2, Max depth = 100, and k-score = 2.

### Gene Set Enrichment Analysis

By taking advantage of ESCC data downloaded from the TCGA project, we carried out GSEA to uncover the signaling pathways and biological processes underpinning the development of ESCC. Patients with ESCC were divided into two groups by the median of UBE2T transcript levels. The data were then prepared according to the guidelines in the website enriched (http://software.broadinstitute.org/gsea/). The GSEA was performed as described previously [18].

### Development of the Prognostic Gene Signature

As previously published [18, 19], we first utilized the least absolute shrinkage and selection operator (LASSO) Cox regression method to generate multivariable models with UBE2T-associated genes. R software 3.6.0 (https://www.r-project.org/) was used to perform the analysis. Briefly, the “glmnet” package for R was used to determine the best model by maximizing model performance with the fewest number of genes. Genes with zero coefficients in the LASSO regression model were removed. Each patient was designated a risk score, which was derived based on the following formula: risk scores $=\sum_{\mathrm{j=}1}^{n}{\text{Coefj*Xj; Coef}}_{\text{j}}$ represented the coefficient, and X_j_ represented the relative expression levels of each gene. The TCGA–ESCC cohort was split into two groups by using the median risk score as a cutoff value. We also used the stepwise Cox regression analysis to optimize the prognostic gene signature. A nomogram was drawn using the rms package for R software [18, 19]. Finally, we accessed the half inhibitory concentration (IC50) of common administrating chemotherapeutic drugs in the TCGA–ESCC cohort using the pRRophetic package [20, 21], based on tumor gene expression levels.

### Statistical Analysis

SPSS 19.0 (IBM. Armonk, NY, United States) and Graphpad Prism 7.0 (GraphPad Software, Inc. San Diego, CA, United States) software was used for statistical analyses. Survival curves were plotted using the Kaplan-Meier method and were compared between the groups using the log-rank test. Both univariate and multivariate Cox analyses were conducted. Significant variables in univariate analysis were selected for multivariate analysis. A *p*-values < 0.05 were considered statistically significant.

## Results

### Elevated Expression *UBE2T* in ESCC

**FIGURE 1:**
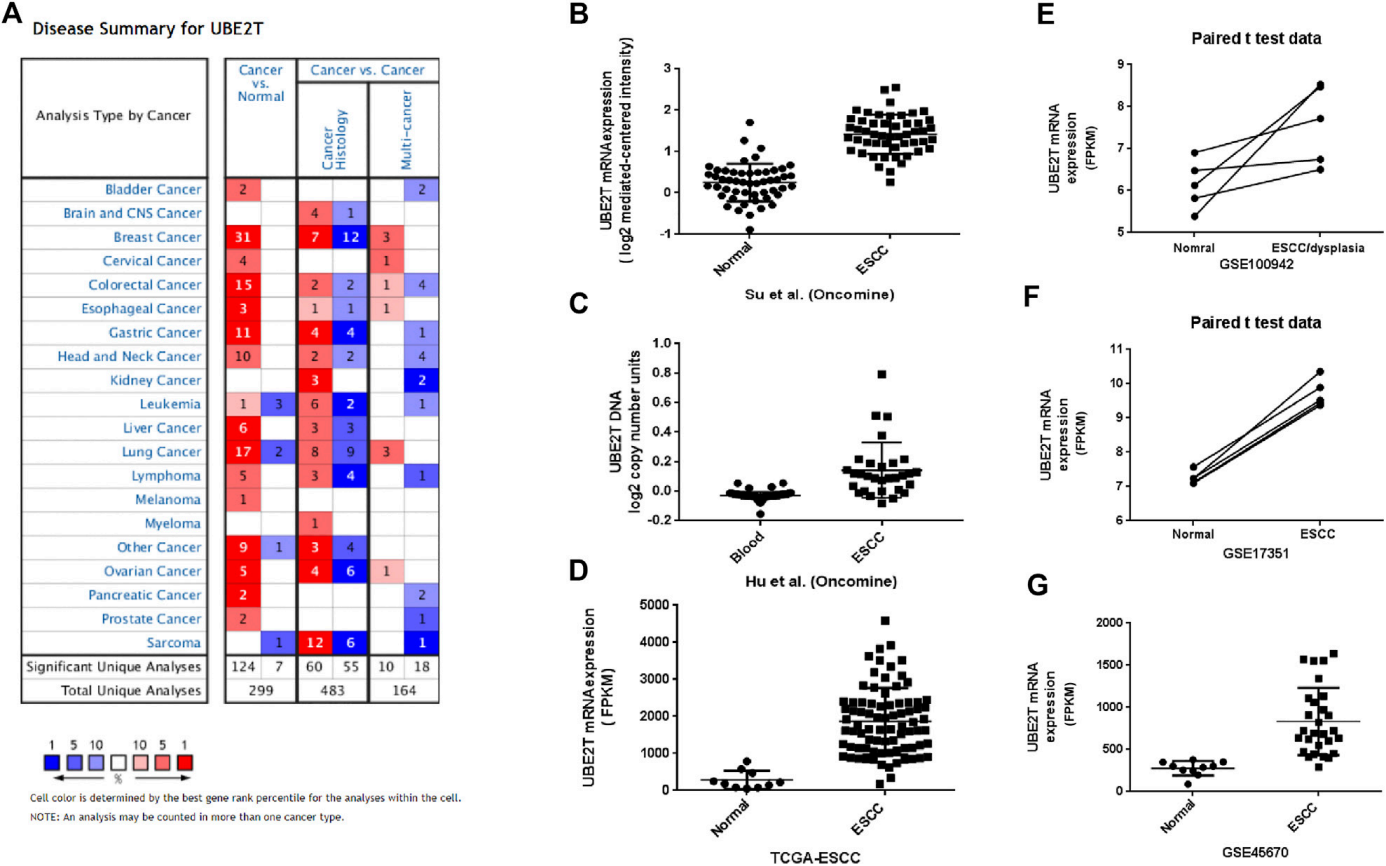
Upregulation of *UBE2T* in ESCC. The overview of studies including *UBE2T* in the Oncomine database **(A)**. A significant increase in the UBE2T DNA copy number in ESCC **(C)**. Significantly elevated mRNA expression levels of the *UBE2T* gene in ESCC when compared with normal tissues in the individual studies from the Oncomine **(B)**, TCGA **(D)**, and GEO database **(E–G)**.

The workflow of the study was shown in Supplementary Figure S1. We first compared the expression of *UBE2T* in esophageal cancer and normal tissues by mining the Oncomine database. After searching the website with “*UBE2T*”, we acquired a summary of studies with significant results for *UBE2T* (*p* < 0.001) in different types of cancer (Figure 1A). We retrieved a total of 4 studies on esophageal cancer involving cancer vs. normal tissues (Table 1). The mRNA expression levels of *UBE2T* in ESCC were significantly enhanced in one study by Su et al. [17]. They reported a 2.243-fold increase in the UBE2T expression in the ESCC (*N* = 51) when compared to the normal esophagus (*N* = 51) (*p* < 0.0001) (Figure 1B). Consistently, Hu et al. found a significant *UBE2T* DNA copy number gain of 1.125 folds in ESCC (Figure 1C) [22]. Additionally, Hao et al. demonstrated a 9.994-fold increase in *UBE2T* transcripts in esophageal adenocarcinoma (Table 1) [23]. Kim et al. reported a similar UBE2T expression tendency in esophageal adenocarcinoma (Table 1) [24].

**TABLE 1:** Studies retrieved from the Oncomine database regarding the comparison of UBE2T between esophageal carcinoma and normal tissue.

UBE2T	Cancer/normal	Fold change	*p* Value	Sample size	References
mRNA	Esophagus/esophageal squamous cell carcinoma	2.243	8.03E−23	51/51	Su
DNA copy number gain	Esophageal squamous cell carcinoma/blood	1.125	1.46E−5	30/102	Hu
mRNA	Esophageal adenocarcinoma/esophagus/Duodenum	9.994	1.24E−4	5/15/11	Hao
mRNA	Esophageal adenocarcinoma/esophagus	1.247	0.024	28/15	Kim

We next used the TCGA database to validate the differential expression of *UBE2T* between esophageal cancers and normal tissues. TCGA–ESCA project consists of both esophageal adenocarcinomas and squamous cell neoplasms. We extracted data on 10 normal tissues and 80 squamous cell neoplasms from the TCGA–ESCA projects and verified significantly enhanced *UBE2T* expression in ESCC (Figure 1D). Moreover, we obtained three GEO datasets on ESCC: GSE100942 (5 pairs of ESCC tumor and adjacent non-tumor tissues), GSE17351 (5 pairs of ESCC tumor and normal tissues), and GSE45670 (38 ESCC tumor tissues and normal esophageal epithelia). Significantly increased expression levels of *UBE2T* in ESCC were also observed in these datasets (Figures 1E–G–G). These results indicate that the UBE2T may be an oncogene in ESCC.

### Signaling Pathways and Cellular Processes Related to UBE2T

Previous studies have reported that UBE2T could affect critical cellular events in different types of cancer, including breast cancer, hepatocellular carcinoma, and multiple myeloma [13–15, 25–28]. With this in mind, we attempted to investigate the UBE2T downstream signaling pathway in ESCC. We first extracted 220 genes correlated with UBE2T (correlation coefficient ≥0.68) from Su’s dataset in the Oncomine. Coexpression genes were then uploaded into an online STRING database to generate a protein–protein interaction (PPI) network. A PPI network of 211 nodes, 4,800 edges were created with an average node degree of 45.5 (Data not shown). The average local clustering coefficient was 0.655. This PPI network has significantly more interactions than what a random set of proteins of similar size would do, indicating that these molecules may be partially biologically connected as a group.

**FIGURE 2:**
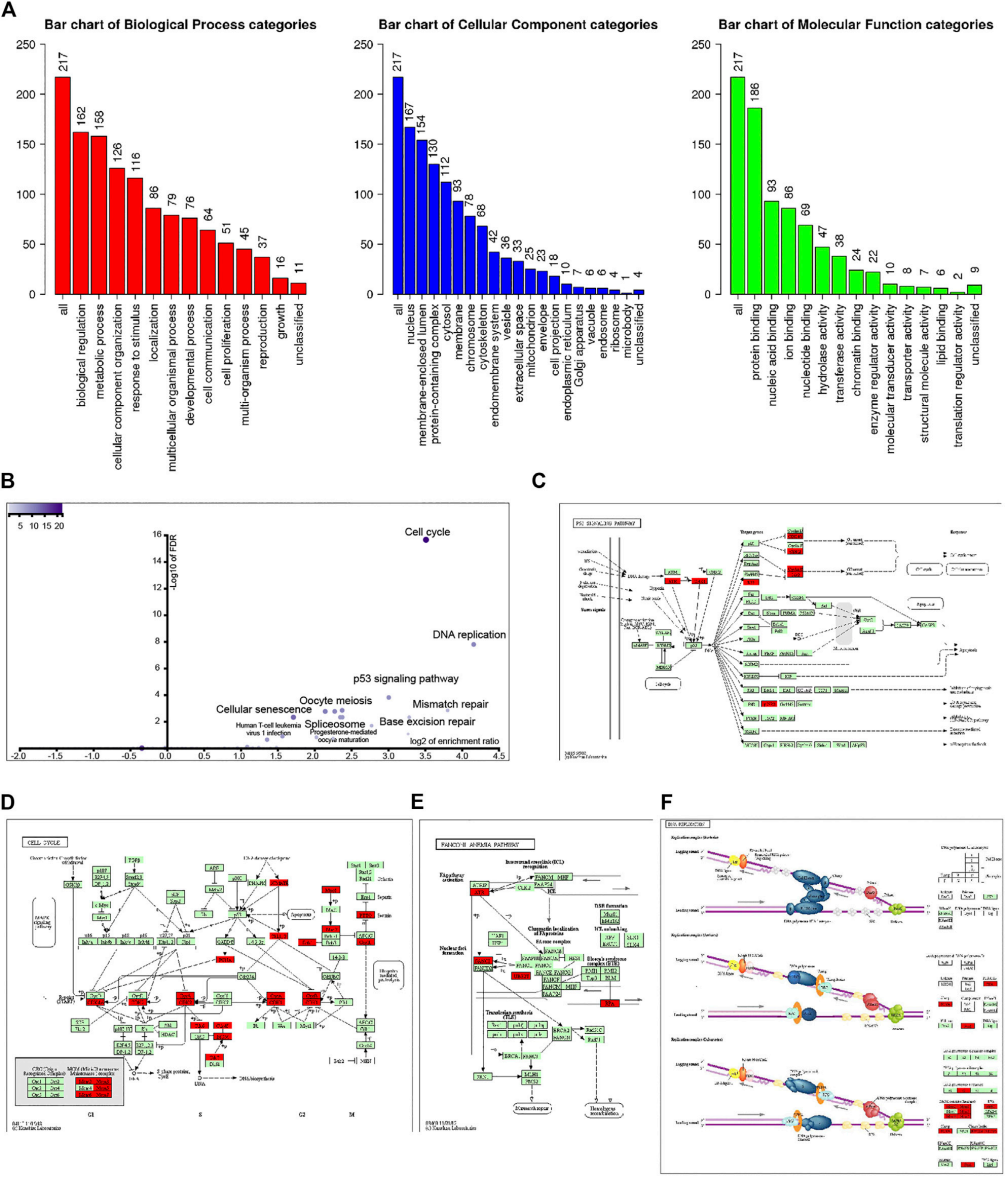
Analysis of 221 UBE2T-related genes (correlation coefficient ≥0.68). GO annotation of these genes **(A)**. KEGG pathway analysis **(B)**. KEGG mapper validated that these UBE2T-related genes were enriched in the p53 signaling pathway **(C)**, cell cycle **(D)**, Fanconi anemia pathway **(E)**, and DNA replication **(F)**.

Go enrichment was used to annotate these genes based on biological processes (BP), cellular components (CC), and molecular functions (MF). Regarding BP, genes coexpressed with UBE2T mainly fall into biological regulation, response to stimulus, cell communication, cell proliferation, growth. MF of these genes was enriched in protein binding, nucleic acid binding, nucleotide binding, chromatin binding, and enzyme regulator activity (Figure 2A).

KEGG pathway analysis further revealed that coexpression genes mainly enriched in the cell cycle, DNA replication, p53 signaling pathway, mismatch repair, pyrimidine metabolism, base excision repair, nucleotide excision repair, purine metabolism, and Fanconi anemia pathway (Figure 2B). We entered the 220 genes into the KEGG Mapper (www.kegg.jp/kegg/mapper.html). These genes (red rectangle) were mainly enriched in the p53 signaling pathway, cell cycle, Fanconi anemia pathway, and DNA replication (Figures 2C–F).

**FIGURE 3:**
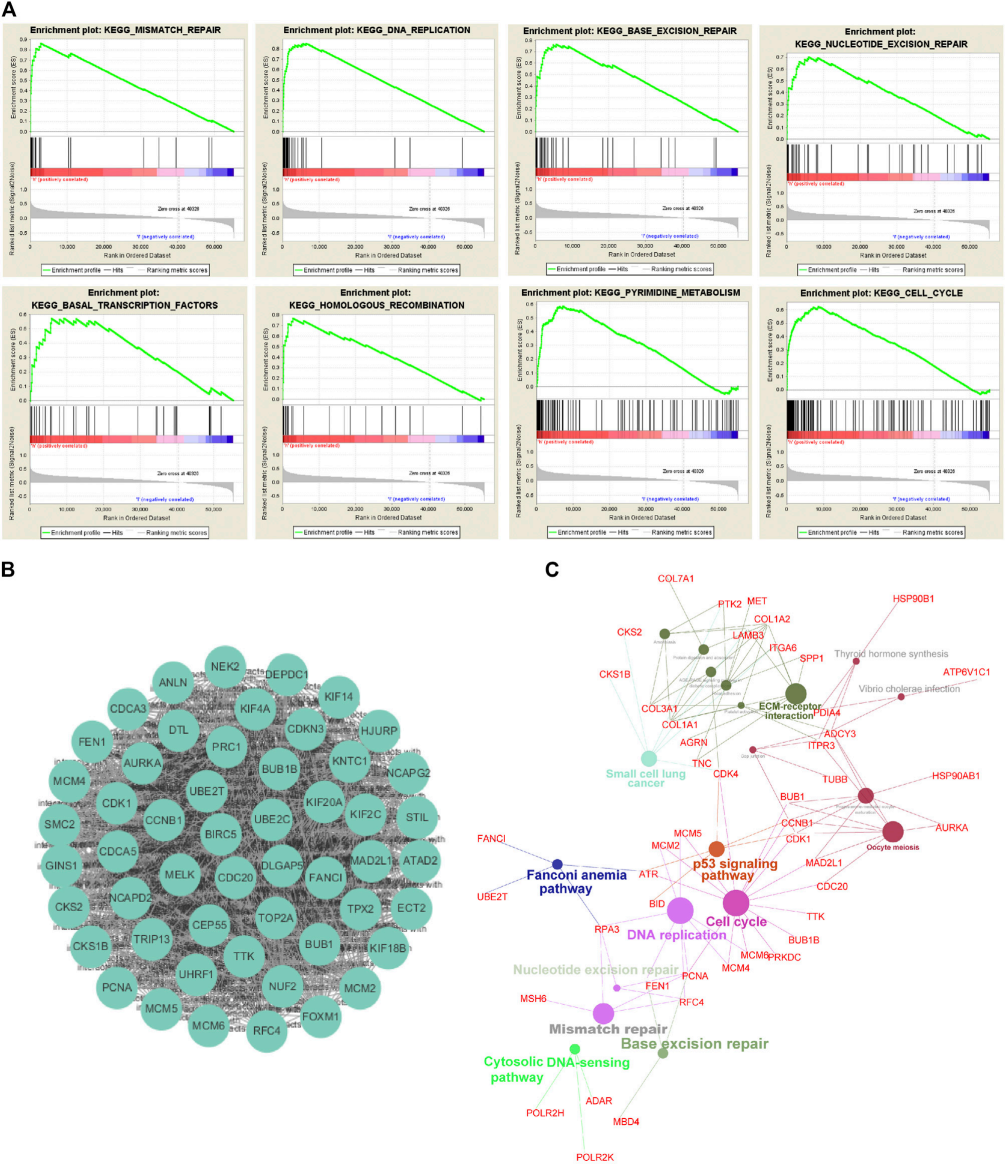
Exploration of UBE2T downstream signaling pathways. Signaling pathways and cellular processes associated with UBE2T, as revealed by gene set enrichment analysis (GSEA) of gene expression data of TCGA-ESCC **(A)**. Hub genes extracted from the top 1% overexpressed genes (177) in ESCC (An Oncomine dataset: Su’s study) **(B)**. Signaling pathways involved in ESCC, as uncovered by KEGG pathway enrichment analysis of the overexpressed genes **(C)**.

Furthermore, we divided TCGA-ESCC patients into two subgroups (*UBE2T* ^high^ vs. *UBE2T* ^low^), using the median UBE2T mRNA level as a cutoff value. And then, we performed GSEA to interrogate the signaling pathways and cellular processes that were significantly associated with the *UBE2T* ^high^ subgroup compared with the *UBE2T* ^low^ subgroup. As shown in Figure 3A, we found that genes upregulated in the *UBE2T* ^high^ subgroup were mostly enriched in the mismatch repair (NES = 2.278, *p* < 0.0001), DNA replication (NES = 2.155, *p* < 0.0001), BER (NES = 2.212, *p* = 0.002), NER (NES = 2.168, *p* = 0.002), basal transcription factors (NES = 1.99, *p* = 0.002), homologous recombination (NES = 2.044, *p* = 0.002), pyrimidine metabolism (NES = 2.116, *p* = 0.006), and cell cycle (NES = 2.061, *p* = 0.015).

### Signaling Pathways in the ESCC

To interrogate signaling pathways responsible for the development of ESCC, we retrieved the top 1% overexpressed genes 177) in ESCC compared to normal tissues from Su’s ESCC study [17]. The MCODE plugin of the Cytoscape revealed that UBE2T was among the most significant molecular module of these top genes (Figure 3B). The ClueGo plugin of the Cytoscape was used to analyze in which KEGG pathways these genes are distributed. Similar to the above presented UBE2T-associated pathways, the essential cellular processes and pathways included DNA replication, cell cycle, mismatch repair, base excision repair, Fanconi anemia pathway, and p53 signaling pathway (Figure 3C). These results verify that UBE2T may play a crucial role in the development of ESCC.

### Development of Prognostic Signature Based on UBE2T and Its Coexpressed Genes

**FIGURE 4:**
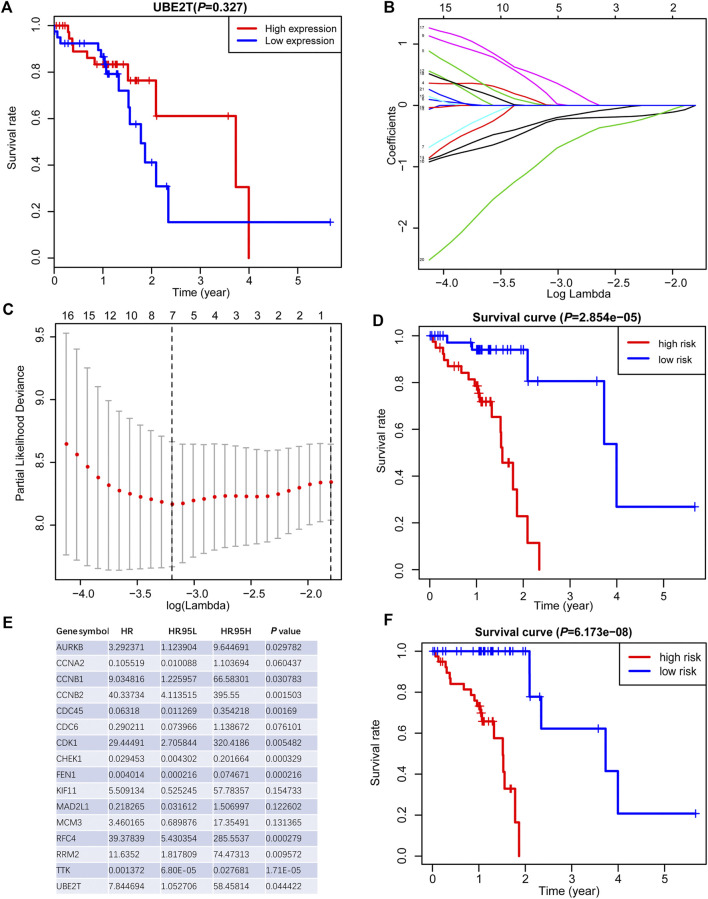
Generation of a prognostic signature from UBE2T-associated genes in the TCGA–ESCC, using the LASSO Cox regression algorithm. Kaplan-Meier curves were plots regarding overall survival (OS) based on mRNA expression levels of *UBE2T*, stratified by median **(A)**. LASSO coefficients of the UBE2T-associated genes in ESCC **(B)** and the identification of the best parameter (lambda) in the LASSO model **(C)**. Kaplan-Meier plots for OS, stratified by the median of risk scores calculated from a 7-gene prognostic signature **(D)**. The list of 16 genes included in the predictive modes by the stepwise Cox regression model and hazard ratio (HR) of each gene derived from univariate Cox regression **(E)**. Kaplan–Meier OS curves, with ESCC patients dichotomized by risk scores **(F)**.

We also tested the association between *UBE2T* transcripts and OS in the TCGA-ESCC cohort and OSescc database (https://bioinfo.henu.edu.cn/DBList.jsp) [29]. However, the mRNA expression levels of the *UBE2T* gene alone were not sufficient to divide patients into subgroups with significantly different OS (Figure 4A, Supplementary Figure S2). With the widespread use of transcriptome sequencing, multiple-gene signatures have emerged as robust biomarkers to predict prognosis in cancers. Therefore, we aimed to develop UBE2T-related gene signatures to predict prognosis in ESCC. Using the Cytoscape plugin Cytohubb, we extracted the top 20 hub genes from 221 genes most correlated with *UBE2T* in Su’s study [17]. We first tried to use the LASSO Cox regression algorithm to construct a prognostic gene signature for the TCGA-ESCC cohort with these hub genes and *UBE2T*. This method yielded a 7-gene prognostic signature (Figures 4B,C). The risk scores calculated based on this gene signature could significantly separate ESCC patients into high-risk and low-risk subgroups in terms of OS. Patients at high risk had significantly shorter lifespans than those at low risk (log-rank test, *p* = 2.854e−05) (Figure 4D).

**FIGURE 5:**
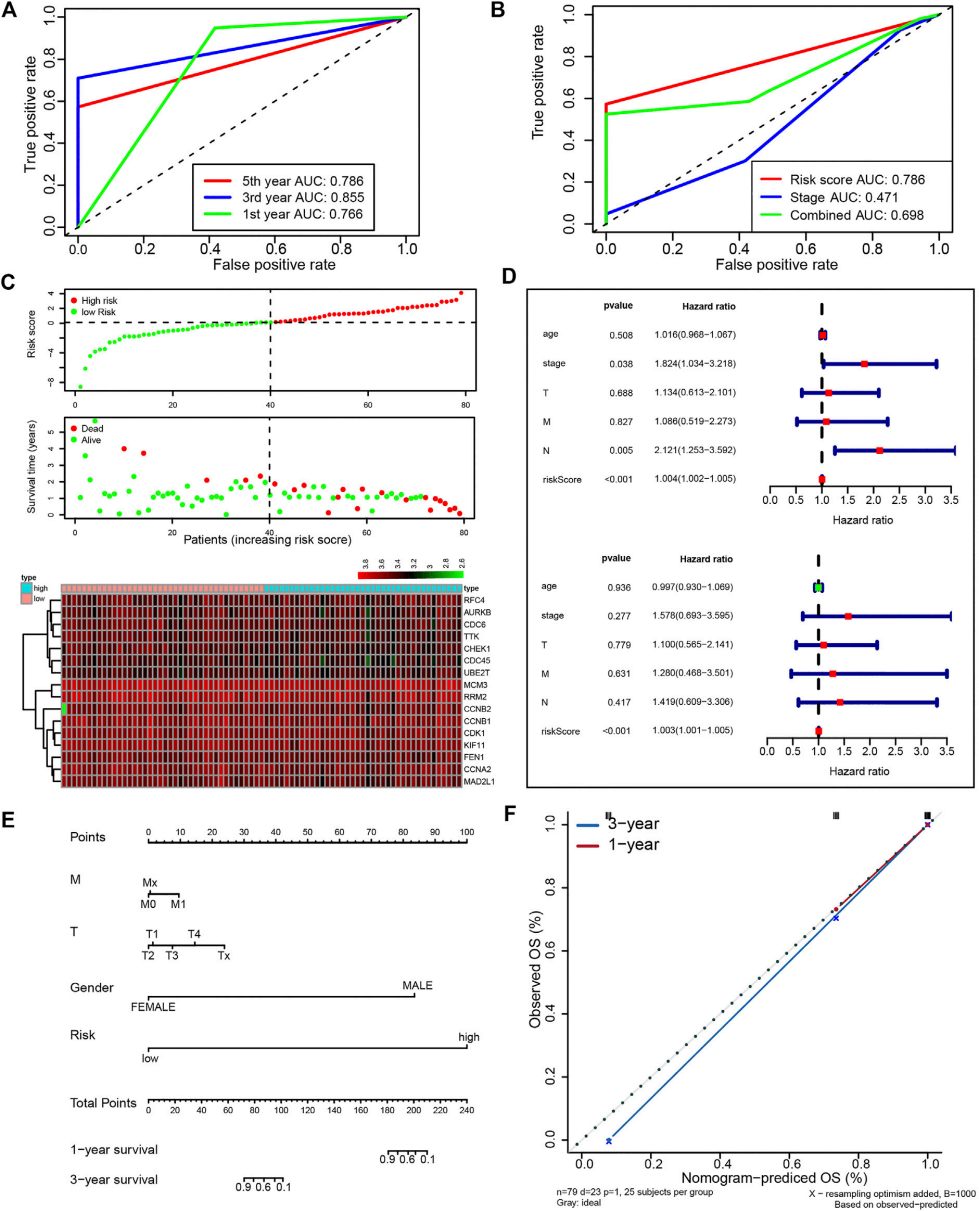
A prognostic signature from 16 UBE2T-associated genes in the TCGA–ESCC patients, established using the stepwise Cox regression model. The risk score was calculated for each patient. The time-dependent ROC of risk scores **(A)** and ROC of variables as indicated **(B)**. Evaluation of the prognostic values of risk scores from the 16-gene signature in TCGA-ESCC. The outline of risk score and corresponding survival status for individual ESCC patients and heatmaps of the 16 gene expression profiles **(C)**. Univariate (upper panel) and multivariate (lower panel) COX regression analysis in ESCC **(D)**. The nomogram integrating the risk score and clinical characteristics of the TCGA-ESCC cohort **(E)**. Calibration curves for 1- and 3-year survival **(F)**.

Next, we attempted to optimize the prognostic model using the stepwise Cox regression analysis. A total of 16 genes were included in the model (Figure 4E). As demonstrated in Figure 4F, the risk scores based on the 16-gene signature could significantly stratify ESCC patients regarding OS, and an even smaller *p*-value was reached (log-rank test, *p* = 6.173e−08). Receiver operating characteristic (ROC) curves were plotted to evaluate the risk scores’ prognostic accuracy, and 1-, 3-, and 5-year area under curve (AUC) values of the risk score were 0.765 and 0.855, 0.786, respectively (Figure 5A). Moreover, the AUC of the risk score was larger than that of the clinical-stage (0.786 vs. 0.471) in Figure 5B. These results demonstrate a decent prognostic performance of the risk score. The heatmap in Figure 5C delineated expression profiles of the 16 genes in ESCC patients. In Figure 5D, the univariate (the upper panel) and multivariate (the lower panel) COX regression analysis showed that the risk scores were associated with survival and independently predicted prognosis in ESCC. The relatively small hazard ratio (HR) may be attributed to the small sample size of the TCGA-ESCC cohort. The nomogram’s C-index was 0.845 (Figures 5E,F), indicating the robustness of the prognostic signature.

### Immunohistochemistry Staining of UBE2T in ESCC Patients and Survival Analysis

**FIGURE 6:**
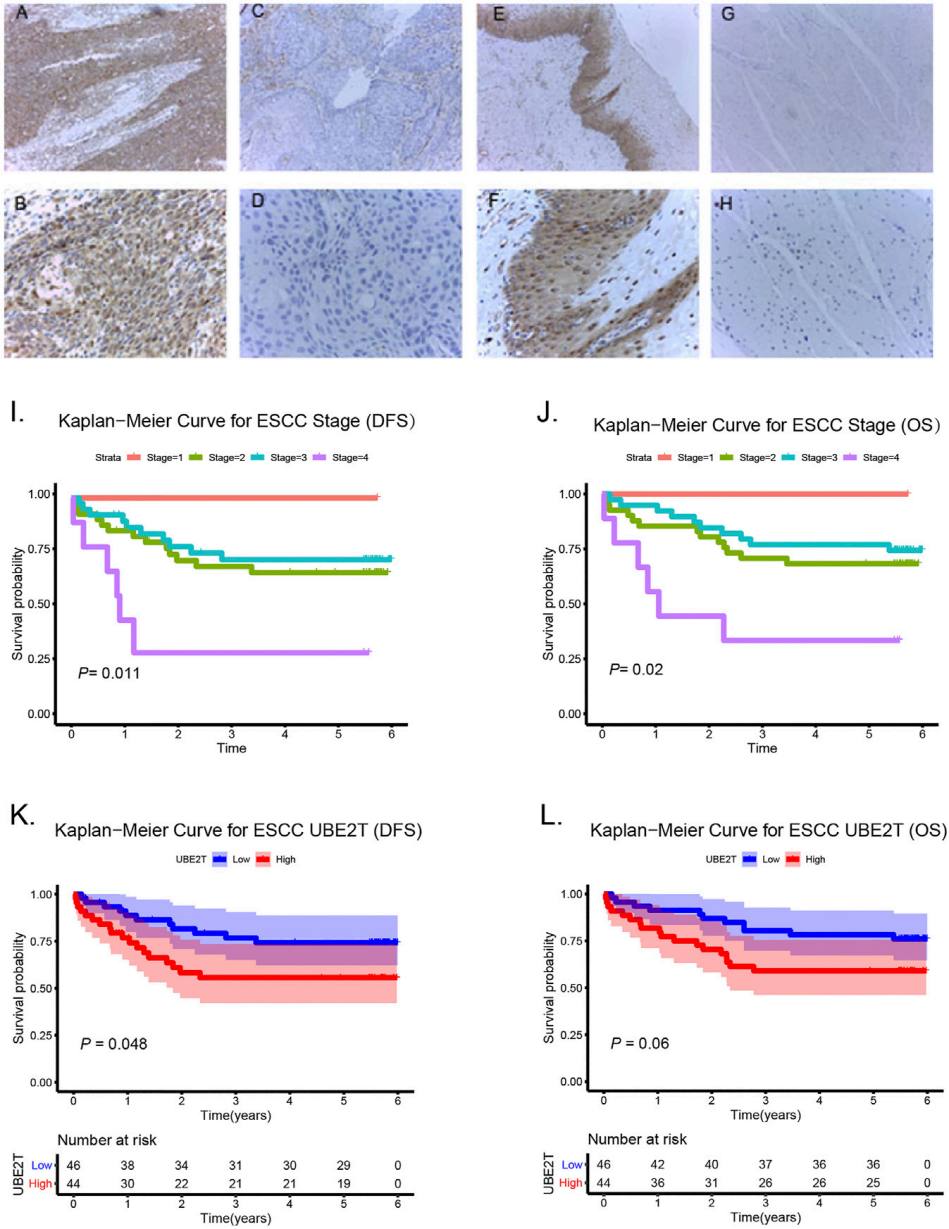
Association of UBE2T expression and prognosis in ESCC (*n* = 90). Immunohistochemical staining of UBE2T in ESCC and adjacent non-cancerous tissues **(A–H)**. High expression of UBE2T in ESCC tissues **(A**,**B)**; Low expression of UBE2T in ESCC tissues **(C**,**D)**; High expression of UBE2T in adjacent non-cancerous tissues **(E**,**F)**; Low expression of UBE2T in adjacent non-cancerous tissues **(G,H)** (**A, C, E, G** 100×; **B, D, F, H** 400×). Kaplan-Meier survival plots for clinical stages **(I**,**J)**. Kaplan–Meier survival plots for immunostaining scores of UBE2T **(K,L)**.

**FIGURE 7:**
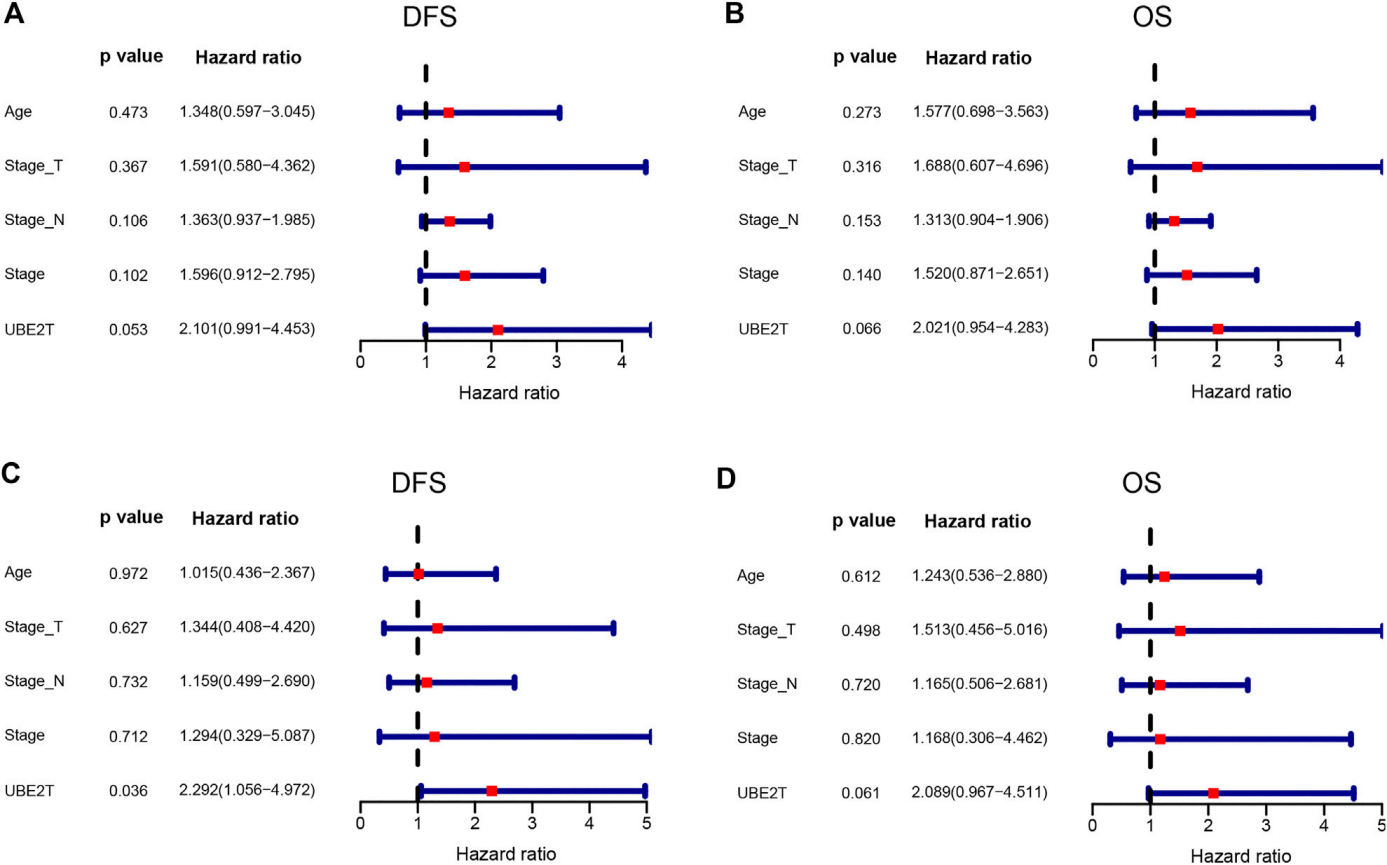
Cox proportional hazards regression analysis. Univariate (upper panel) and multivariate (lower panel) COX regression analysis in ESCC.

In this study, we also enrolled 90 ESCC patients to investigate the role of UBE2T in ESCC, who received surgery in our hospital (Supplementary Table S1). ESCC and peritumoral tissue samples were collected after surgery. We conducted immunohistochemical staining to evaluate the expression of UBE2T in ESCC samples. The positive UBE2T immunostaining was mainly observed in the cytoplasm of cells (Figures 6A–H). UBE2T immunostaining was scored from 0 to 4. ESCC tissues with immunostaining scores ≤1 or ≥2 were categorized into UBE2T^low^ and UBE2T^high^ groups, respectively. The association of the clinical-stage with disease-free survival (DFS) and overall (OS) of ESCC patients were shown in Figures 6I,J. Kaplan–Meier survival analysis demonstrated that UBE2T protein levels were significantly associated with DFS but not OS of ESCC patients. Patients with UBE2T^low^ tumors exhibited significantly longer DFS than those with tumors (Figures 6K,L). Univariate and multivariate Cox regression analysis was also performed (Figure 7).

### Association of the Risk Model With Chemotherapy Sensitivity

**FIGURE 8:**
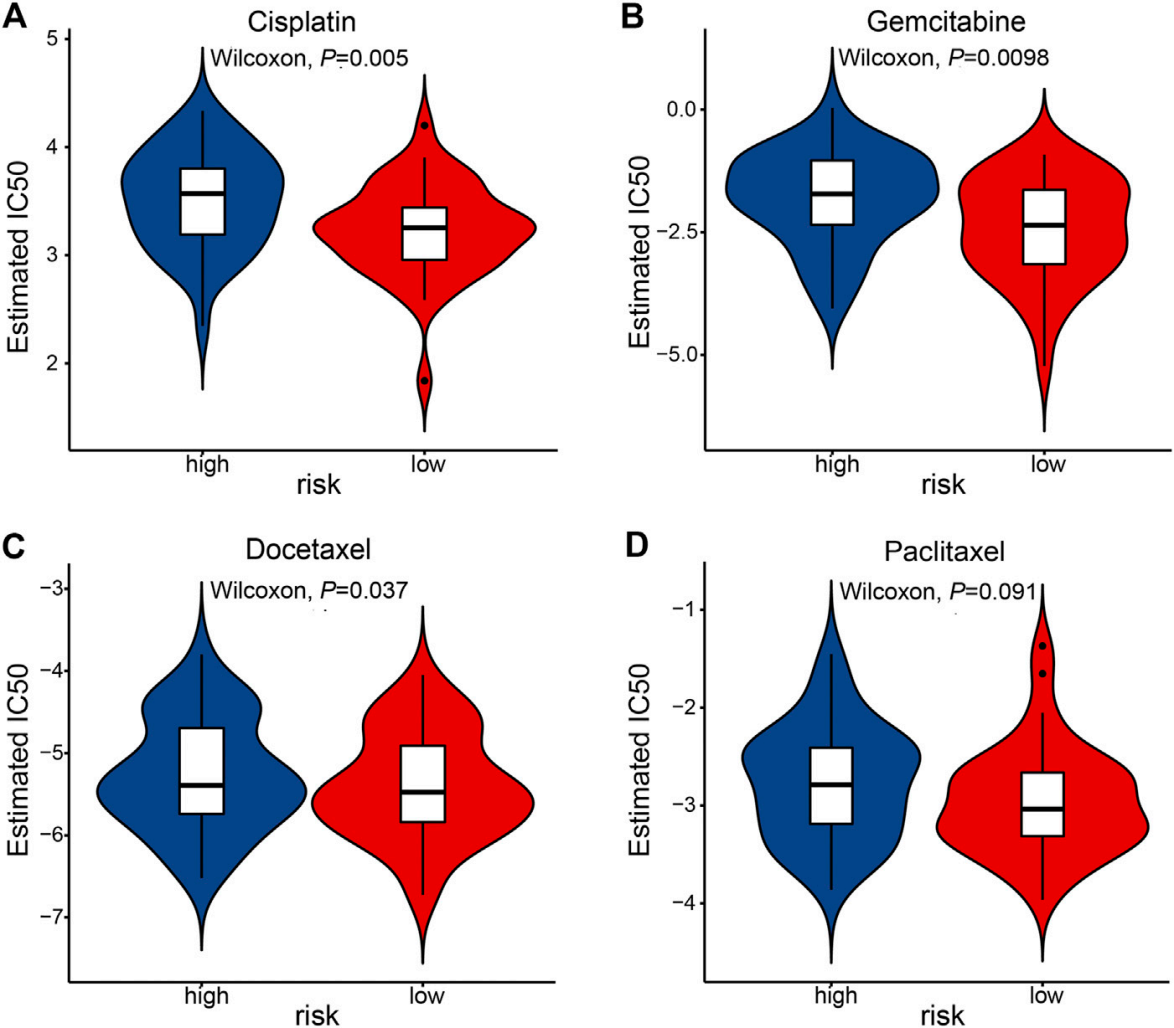
The association of prognostic signature and response to chemotherapy in the TCGA-ESCC cohort. IC50 was calculated for cisplatin **(A)**, gemcitabine **(B)**, docetaxel **(C)**, and paclitaxel **(D)**.

Last, we tested whether the prognostic signature is associated with ESCC patients’ response to standard chemotherapy. An R package pRRophetic [20, 21] allowed us to calculate the IC50 of common chemotherapeutic drugs in the TCGA-ESCC cohort, including cisplatin, paclitaxel, gemcitabine, docetaxel. IC50 values suggested that ESCC patients in the low-risk group were significantly more sensitive to cisplatin and gemcitabine (Figure 8).

## Discussion

Esophageal cancer has been one of the serious global public health problems, with ESCC ranked as the sixth leading cause of cancer-related death. Tumorigenesis of ESCC is a multi-step and complicated process involving genomic instability, driver gene mutation, resistance to death signals, and cell signal transduction dysfunction [30]. Despite the remarkable progress made in genetics and molecular biology of ESCC, its pathogenesis has not been fully elucidated. It is imperative to identify novel critical druggable targets and elucidate potential mechanisms underpinning the development of ESCC.

In this study, we explored the clinical relevance and potential mechanisms of UBE2T in ESCC. UBE2T is closely related to the tumorigenesis and progress of various cancer, and its oncogenic potential is attracting more and more attention. Preferential expression of UBE2T in cancerous tissues to adjacent non-cancerous tissue has been found in a broad spectrum of cancers [11, 13, 16, 28, 31]. By screening the ubiquitin pathway genes, Perez-Peña et al. found that UBE2T expression levels were significantly higher in basal-like breast cancers than in normal breast tissues, and the increased UBE2T expression was significantly associated with an unfavorable prognosis [11]. Liu et al. demonstrated significantly increased UBE2T in HCC at both the mRNA and protein levels compared with non-tumor tissues [13]. Besides solid tumors, the linkage between high UBE2T and poor survival has been recently reported in multiple myeloma [31]. However, the role of UBE2T in ESCC has not been reported. By mining several datasets from the Oncomine, TCGA, and GEO, we demonstrated significant increases in *UBE2T* transcripts in ESCC compared to normal tissues. Consistently, significantly elevated *UBE2T* gene copy number in ESCC was also observed in a cohort. More importantly, with immunohistochemistry staining, we found that the UBE2T expression levels were inversely associated with DFS in ESCC. Our findings were in line with previous studies. Therefore, it is reasonable to speculate that UBE2T may participate in and promote the development of ESCC.

Apart from clinical significance, functional analyses have suggested an oncogenic role of UBE2T [5, 12, 14, 32]. For instance, UBE2T stimulated the proliferation and invasion of cancer cells through the PI3K/AKT pathway in osteosarcoma cells [15]. Gong and colleagues observed that the knockdown of *UBE2T* inhibited the proliferation of bladder cancer cells and led to cell cycle arrest and apoptosis [32]. Similarly, UBE2T was reported to accelerate liver cancer cells’ growth by facilitating the ubiquitination and degradation of p53 in HCC [13]. Reversely, the depletion of UBE2T in bladder cancer suppressed tumor growth and concomitantly induced cell cycle arrest and apoptosis [32]. In prostate cancer, UBE2T mediated the proliferation and epithelial-mesenchymal transition (EMT) of prostate cancer cells by regulating vimentin [16]. UBE2T was also observed to promote breast cancer cell proliferation by specifically regulating the ubiquitination-mediated degradation of breast cancer-associated protein 1 (BRCAl) [25]. Given the involvement of UBE2T in various tumors, drugs targeting this molecule have been developed [5, 33]. For instance, Morreale et al. discovered several low molecular weight compounds that can bind to UBE2T through a biophysical fragment screening, known as fragment-based drug discovery [33]. These compounds could inhibit substrate ubiquitination activity of UBE2T [33].

Accordingly, mechanisms underpinning the tumor-promoting roles of UBE2T have been intensively investigated. UBE2T was initially identified as an E2 ubiquitin-conjugating enzyme in the Fanconi anemia pathway responsible for efficiently repairing damaged DNA [34]. UBE2T-depleted cells exhibited defective DNA repair capacity [34]. In breast cancer, UBE2T was shown to facilitate the polyubiquitination and degradation of BRCA1—an E3 ubiquitin ligase and a critical tumor suppressor gene in hereditary breast cancer--by interacting with the BRCA1/BRCA1-associated RING domain protein (BARD1) complex [25]. Another evidence shows that UBE2T directly promotes nucleotide excision repair (NER), while the knockdown of *UBE2T* impairs cells’ capacity of removing UV-induced DNA damages [27].

In the current study, by performing KEGG pathway enrichment analysis with UBE2T-associated genes in the study Su et al. [17], we revealed that UBE2T was associated with cell cycle, DNA replication, p53 signaling pathway, cellular senescence, mismatch repair, and base excision repair. We also validated these findings by conducted GSEA with the ESCC dataset in TCGA. Our results were consistent with the previous reports. It suggests that UBE2T may promote the development of ESCC by regulating these cellular activities and signaling pathways. *In vivo* and *in vitro* experiments should be conducted to validate the functions of UBE2T in ESCC in the future.

As we mentioned above, The prognostic values of UBE2T have been studied in lung and breast cancer [11], HCC [13], and multiple myeloma [31]. In agreement with previous studies, we found that high expression levels of UBE2T were associated with unfavorable DFS, as unveiled by the Kaplan-Meier survival analysis. Moreover, the emerging of high throughput technologies, such as next-generation sequencing, has led to prognostic signatures with multiple gene expression profiles. Great success has been achieved in predicting clinical outcomes by various types of multi-gene signatures, including immune gene signatures, m6A regulatory gene signatures, and autophagy gene signatures [18, 19, 35–38]. We also developed a gene signature with UBE2T-related genes, which classified ESCC patients into two groups with significantly different OS. These results further confirmed the prognostic promise of UBE2T in ESCC.

Cisplatin, paclitaxel, gemcitabine, docetaxel are regular chemotherapeutic agents for treating ESCC patients. However, only a fraction of ESCC response to chemotherapy. A biomarker predicting chemotherapy sensitivity will facilitate physicians to select patients who are more suitable for chemotherapy. Using an R package pRRophetic [20, 21], we found that ESCC patients in the low-risk group were significantly more sensitive to cisplatin and gemcitabine. The R package’s robustness in predicting response to chemotherapies has been demonstrated in different clinical trials [20].

However, it should be noted that there are some limitations to the current study. First, the sample size of this study is relatively small. As a result, statistical power may be limited. More patients should be recruited in future studies. Second, *in vitro* and *in vivo* studies should be carried out to validate the impacts of UBE2T on the development of ESCC. Third, the molecular mechanisms by which UBE2T contributes to ESCC should be explored. Finally, It is essential to validate the differential expression of UBE2T and its 16 related genes in normal esophageal cells and ESCC cells.

In summary, our results provided evidence of the involvement of UBE2T in ESCC by revealing the prognostic values of UBE2T and potentially affected signaling pathways. UBE2T may serve as a potential target for the treatment of ESCC.

## Data Availability

The original contributions presented in the study are included in the article/Supplementary Material, further inquiries can be directed to the corresponding author.
