# Supplementary material for: UBE2T Contributes to the Prognosis of Esophageal Squamous Cell Carcinoma
Source: Pathol Oncol Res. 2021 Apr 9;27:632531. doi: 10.3389/pore.2021.632531 (PMC8262217; doi:10.3389/pore.2021.632531)
Supplement: Supplementary file 1 [file Table1.DOCX]

| Supplemental Table 1. Characteristics of ESCC patients. | | | | | |
| --- | --- | --- | --- | --- | --- |
|  | |  | **UBE2T**  **Low Expression** | **UBE2T**  **High Expression** | ***P* Value** |
| Total | 90 | | 46 | 44 |  |
| Age |  | |  |  | 0.10 |
|  | ≤65 | | 39 | 31 |  |
|  | ＞65 | | 7 | 13 |  |
| Stage |  | |  |  | 0.29 |
|  | Stage I | | 0 | 1 |  |
|  | Stage II | | 18 | 23 |  |
|  | Stage III | | 24 | 15 |  |
|  | Stage IV | | 4 | 5 |  |
| Stage_T |  | |  |  | 0.59 |
|  | T1 | | 0 | 1 |  |
|  | T2 | | 4 | 6 |  |
|  | T3 | | 40 | 36 |  |
|  | T4 | | 2 | 1 |  |
| Stage_N |  | |  |  | 0.53 |
|  | N0 | | 17 | 22 |  |
|  | N1 | | 14 | 10 |  |
|  | N2 | | 12 | 8 |  |
|  | N3 | | 3 | 4 |  |
| Stage_M |  | |  |  | 0.83 |
|  | M0 | | 46 | 44 |  |
|  | M1 | | 0 | 0 |  |
