# Supplementary figures and images for: UBE2T Contributes to the Prognosis of Esophageal Squamous Cell Carcinoma
Source: Pathol Oncol Res. 2021 Apr 9;27:632531. doi: 10.3389/pore.2021.632531 (PMC8262217; doi:10.3389/pore.2021.632531)

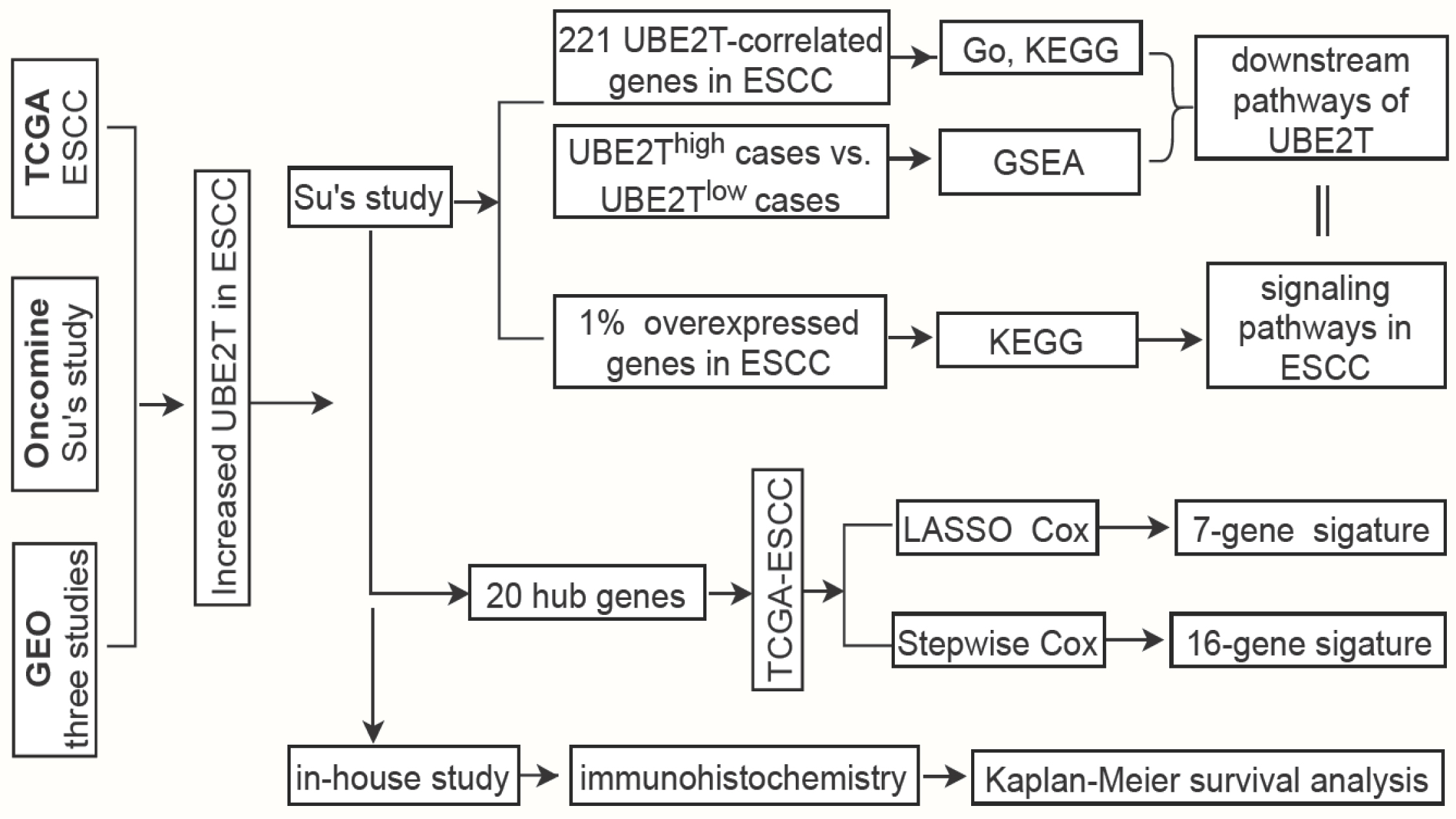

Supplement: Supplementary file 2 [file Image1.JPEG]

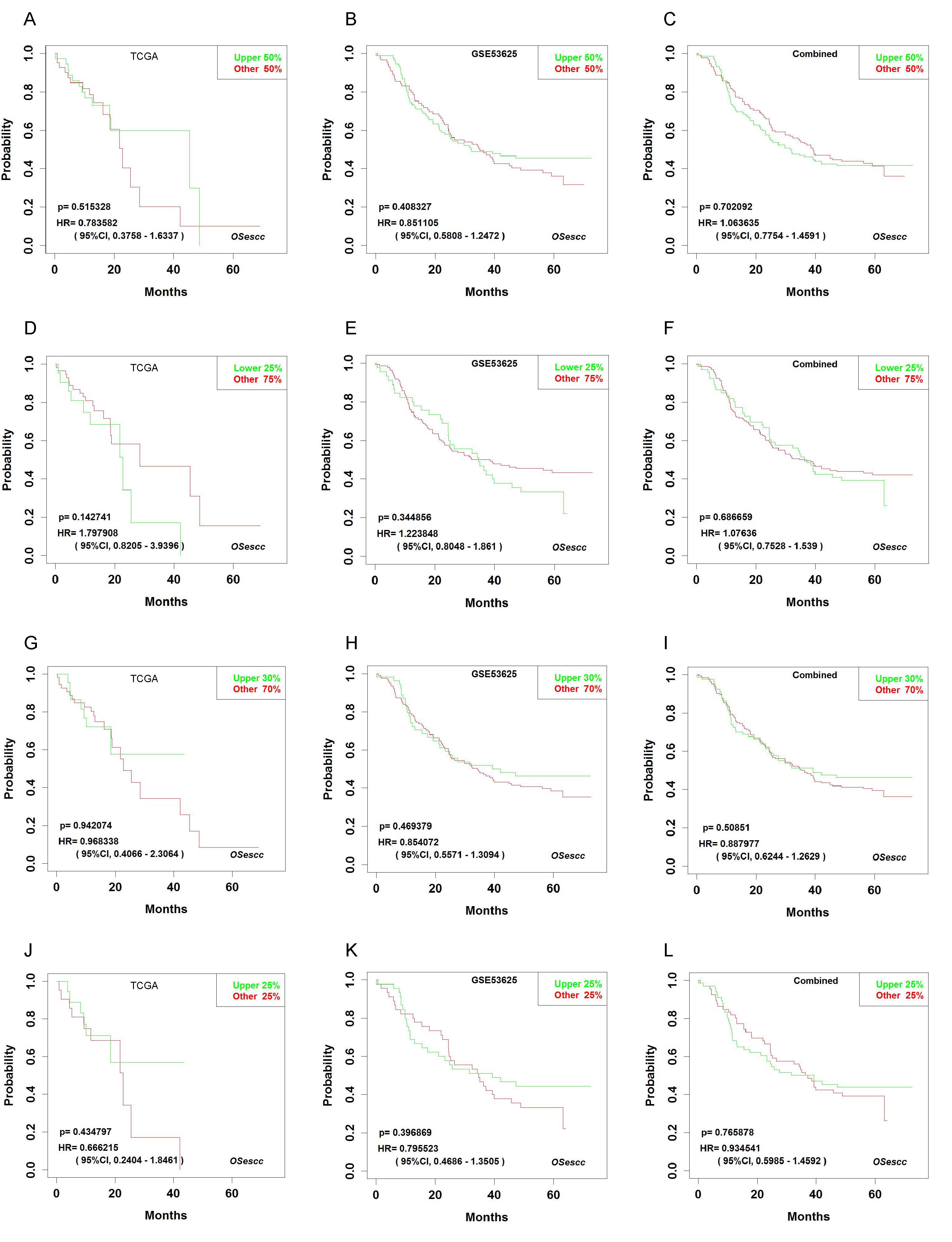

Supplement: Supplementary file 3 [file Image2.JPEG]
